# 1718. *In Vitro* Activity of Imipenem/Relebactam against Class C β-lactamase-Positive Enterobacterales in the Asia/Pacific Region: SMART 2018-2020

**DOI:** 10.1093/ofid/ofac492.1348

**Published:** 2022-12-15

**Authors:** Sibylle Lob, Mark Estabrook, Wei-Ting Chen, Fakhar Siddiqui, Katherine Young, Mary Motyl, Daniel F Sahm

**Affiliations:** Merck & Co., Inc., Schaumburg, Illinois; IHMA, Schaumburg, Illinois; MSD Taiwan, Taipei, Taipei, Taiwan; Merck & Co., Inc., Schaumburg, Illinois; Merck, Rahway, New Jersey; Merck, Rahway, New Jersey; IHMA, Schaumburg, Illinois

## Abstract

**Background:**

Imipenem/relebactam (IMR) is a combination of imipenem/cilastatin with the β-lactamase inhibitor relebactam, an inhibitor of class A and C β-lactamases. We evaluated the activity of IMR and comparators against AmpC- and extended-spectrum β-lactamase (ESBL)-producing *E. coli* and *K. pneumoniae* as well as against isolates of intrinsic AmpC-producing Enterobacterales species that were collected in 9 countries in Asia/Pacific as part of the global SMART surveillance program.

**Methods:**

In 2018-2020, 48 clinical laboratories in Australia, Hong Kong, Malaysia, New Zealand, Philippines, South Korea, Taiwan, Thailand, and Vietnam each collected up to 250 consecutive, aerobic or facultative, gram-negative pathogens per year from patients with bloodstream, intraabdominal, lower respiratory tract, and urinary tract infections. MICs were determined using CLSI broth microdilution and interpreted with 2022 CLSI breakpoints. Isolates that were ertapenem- (2018 only), imipenem-, IMR-, or ceftolozane/tazobactam-nonsusceptible were screened by PCR and Sanger sequencing for β-lactamases.

**Results:**

As shown in the table, IMR maintained activity against ≥96% *K. pneumoniae* and *E. coli* that carried *ampC* with or without ESBL as well as against those intrinsic *ampC* carriers (except *S. marcescens*, 92.7%) among which no additional β-lactamases other than ESBLs were identified. The addition of relebactam increased the susceptibility to imipenem alone by 8-55 percentage points for *K. pneumoniae* and *E. coli* carrying AmpC with or without ESBL. Among intrinsic *ampC* carriers, the largest increase was seen among *K. aerogenes* (28 percentage points). Relebactam restored susceptibility to 97.3% of imipenem-nonsusceptible (NS) *ampC*-positive *K. pneumoniae* (n=73) and to 95.7%, 98.6%, and 37.7% of imipenem-NS isolates of *E. cloacae* complex (n=92), *K. aerogenes* (n=147), and *S. marcescens* (n=61) that carried no acquired β-lactamases. Among the imipenem-NS *S. marcescens* isolates, 50.8% tested with an IMR MIC of 2 µg/mL, which would be susceptible according to EUCAST guidelines.

**Figure:**
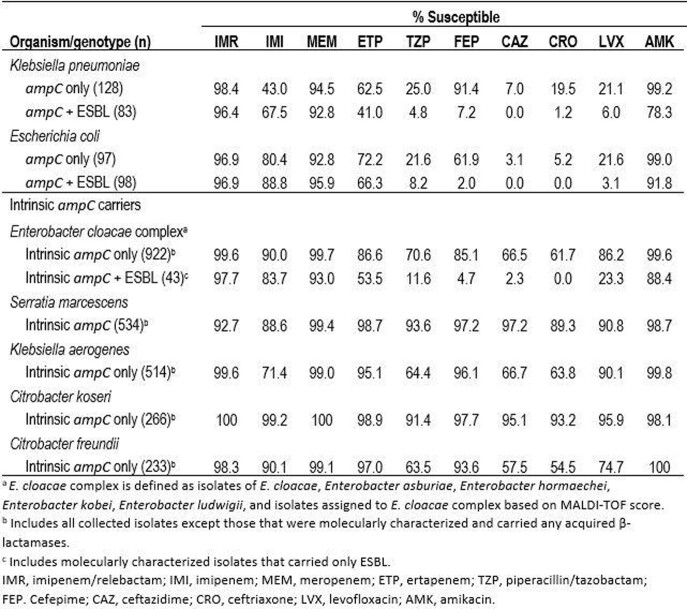

**Conclusion:**

IMR showed strong activity against clinical Enterobacterales isolates that carried either acquired or intrinsic *ampC* with or without ESBL collected in Asia/Pacific.

**Disclosures:**

**Fakhar Siddiqui, MD, MBA**, Merck & Co., Inc.: employee|Merck & Co., Inc.: Stocks/Bonds **Katherine Young, M.S.**, Merck & Co., Inc.: Stocks/Bonds.

